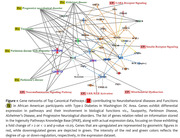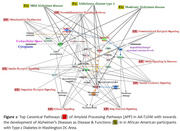# Metabolic Dysfunction and Alzheimer’s Disease Risks in African Americans

**DOI:** 10.1002/alz.086476

**Published:** 2025-01-09

**Authors:** Tanmoy Mondal, Jheannelle Johnson, Tapas Kumar Sur, Christopher A. Loffredo, Sharleine T. Cotin, Jasneet Sahota, Brent E. Korbe, Gail Nunlee‐Blnad, Somiranjan Ghosh Ghosh

**Affiliations:** ^1^ Howard University, Washington, DC USA; ^2^ Howard University, Washington DC, DC USA; ^3^ Georgetown University, Washington DC, DC USA; ^4^ Howard University Hospital, Washington DC, DC USA

## Abstract

**Background:**

Metabolic diseases like chronic Type 2 Diabetes Mellitus (T2DM) are now a serious global health concern In the United States. African Americans (AA) are being affected at a disproportionate rate with the condition compared to other ethnic groups, yet there are relatively few studies that have specifically focused on this group. Our previous findings have suggested that AA patients with T2DM had gene expression signals associated with Alzheimer’s Disease (AD). The current pilot study aimed to examine the amyloidogenic processing pathways (APP) and cholesterol biosynthesis‐related marker genes in the circulation of clinically identified T2DM AA patients in the Washington DC area.

**Method:**

A total of 12 confirmed T2DM AA patients and matched healthy controls were recruited from a regional hospital's outpatient departments, with sociodemographic and medical details obtained for analysis. Whole blood was collected in DNA/RNA Shield™ Blood Collection Tube for RNA extraction and isolation. The APP and cholesterol biosynthesis‐related gene expression patterns were examined using profiler array gene expression (qRT‐PCR). The affected pathways were identified using Ingenuity Pathway Analysis (IPA).

**Result:**

We identified Amyloid Processing, Neuroinflammation Signaling, ERB4 Signaling, Interleukin signaling, and nNos signaling as the top canonical pathways. The top diseases and their functions were Metabolic disease, Neurological disease, Organismal Injury and Abnormalities, Psychological disorders, and cardiovascular diseases. APP, CDK5R1, PSENEN, and MAPT were the key genes that were differentially expressed (≥2‐fold change at p <0.05) in APP pathways, with PHLPP2, IL33, APP, CRP, & TAC1 as the top upstream regulators.

**Conclusion:**

The results suggest that T2DM may influence the risk of developing AD in AA patients. Further studies on larger multi‐ethnic populations would be of interest to validate and extend these findings. Information from such biochemical and genetic studies will be crucial to the development of therapeutic targets to assess future AD risks.